# Investigation into the Effect of Molds in Grasses on Their Content of Low Molecular Mass Thiols

**DOI:** 10.3390/ijerph9113789

**Published:** 2012-10-24

**Authors:** Jiri Skladanka, Vojtech Adam, Ondrej Zitka, Olga Krystofova, Miroslava Beklova, Rene Kizek, Zdenek Havlicek, Petr Slama, Adam Nawrath

**Affiliations:** 1 Department of Animal Nutrition and Forage Production, Faculty of Agronomy, Mendel University in Brno, Zemedelska 1, CZ-613 00 Brno, Czech Republic; Email: adam.nawrath@mendelu.cz; 2 Department of Chemistry and Biochemistry, Faculty of Agronomy, Mendel University in Brno, Zemedelska 1, CZ-613 00 Brno, Czech Republic; Email: vojtech.adam@mendelu.cz (V.A.); zitkao@seznam.cz (O.Z.); olga.krystofova@seznam.cz (O.K.); kizek@sci.muni.cz (R.K.); 3 Central European Institute of Technology, Brno University of Technology, Technicka 3058/10, CZ-616 00 Brno, Czech Republic; 4 Department of Veterinary Ecology and Environmental Protection, Faculty of Veterinary Hygiene and Ecology, University of Veterinary and Pharmaceutical Sciences, Palackeho 1–3, CZ-612 42 Brno, Czech Republic; Email: beklovam@vfu.cz (M.B.); 5 Department of Animal Morphology, Physiology and Genetics, Faculty of Agronomy, Mendel University in Brno, Zemedelska 1, CZ-613 00 Brno, Czech Republic; Email: zdenek.havlicek@seznam.cz (Z.H.); petr.slama@mendelu.cz (P.S.)

**Keywords:** grass, mold, thiols, glutathione, phytochelatin

## Abstract

The aim of this study was to investigate the effect of molds on levels of low molecular mass thiols in grasses. For this purpose, the three grass species *Lolium perenne*, *Festulolium pabulare* and *Festulolium braunii* were cultivated and sampled during four months, from June to September. The same species were also grown under controlled conditions. High-performance liquid chromatography with electrochemical detection was used for quantification of cysteine, reduced (GSH) and oxidized (GSSG) glutathione, and phytochelatins (PC2, PC3, PC4 and PC5). Data were statistically processed and analyzed. Thiols were present in all examined grass species. The effect of fungicide treatments applied under field conditions on the content of the evaluated thiols was shown to be insignificant. Species influenced (*p* < 0.05) PC3 and GSSG content. *F. pabulare*, an intergeneric hybrid of drought- and fungi-resistant *Festuca arundinacea*, was comparable in PC3 content with *L. perenne* and *F. braunii* under field conditions. Under controlled conditions, however, *F. pabulare* had higher (*p* < 0.05) PC3 content than did *L. perenne* and *F. braunii*. Under field conditions, differences between the evaluated species were recorded only in GSSG content, but only sampling in June was significant. *F. pabulare* had higher (*p* < 0.05) GSSG content in June than did *L. perenne* and *F. braunii*.

## 1. Introduction

Worldwide, more than 7500 species of grasses are known, but only a minority of these is used as cultivated species in meadows or pasture crops. Individual species differ not only morphologically (by height, manner of tillering, and inflorescence), but also biochemically, as reflected in the organic content (crude protein, fat, and fiber) and inorganic nutrient (P, K, Na, and Mg) levels. Forage grasses are characterized by their high quality for feeding purposes, as demonstrated by low-fiber elements and higher energy content. As a result of these very characteristics, many of these species show lower resistance to pathogens, and especially molds. Grasses are well adapted to periodic defoliation and reproduce not only sexually, but also vegetatively [1,2,3]. Due to this fact, they do not require specific defense mechanisms against herbivores. Nevertheless, alkaloids have been detected in some species, produced in conjunction with endophytic fungi [4]. Therefore, some protective mechanisms must exist, including synthesis of stress-induced molecules.

Glutathione is one of the most significant thiol compounds occurring in both the plant and animal kingdoms. It can be found in all living organisms, from prokaryotic to eukaryotic. Glutathione protects the cytosol and other cellular parts against reactive oxygen radicals (ROS) induced by biotic and abiotic stress. Glutathione occurs in organisms in two forms—as reduced glutathione (GSH) and oxidized glutathione (GSSG). These two forms are strictly maintained in a certain ratio, disturbance of which is an indicator of stress elicited by various factors [5,6,7,8]. GSSG originates by formation of a disulfide bond between two GSH molecules, wherein the two H+ atoms formed participate in the ascorbate-glutathione cycle towards eliminating generated ROS. Regressive GSSG molecule regeneration proceeds under GSH catalysis by reduction and oxidation of NADPH + H^+^ [9,10]. Its concentration varies in plants within a range of 0.1 to 10 mm [11]. 

The first-ever reference to glutathione is from 1888, when its presence in yeasts was demonstrated. Glutathione’s structure was described as late as 1935. In the 1960s, GSH was studied intensively because of its connection with human body fluids [12,13,14,15]. However, the major contribution to clarifying glutathione metabolism is attributed to Meister [11]. GSH is a tripeptide containing γ-glutamyl-cysteinyl-glycine [16]. In higher plants, GSH plays crucial roles in maintaining cellular redox homeostasis and also participates in detoxification of heavy metals and xenobiotics. In relation to these functions, GSH is also utilized as a signal molecule in cells. GSH/GSSG couple reduction (redox) potential is not only affected by the reciprocal rate of GSH/GSSG but also by changes in GSH synthesis as well as degradation [17,18,19,20,21].

Metallothionein-like proteins can be considered another group of plant protective compounds. These are polypeptides sharing a low molecular mass, high cysteine content with absence of aromatic amino acids and histidine, high metal content, and an abundance of CysXCys sequences, where X is an amino acid other than cysteine [22,23,24,25]. Metallothioneins (MT) are subdivided into three classes based on their structures (Class I: polypeptides with location of cysteine closely related to those in equine renal metallothioneins; Class II: polypeptides with locations of cysteine only distantly related to those in equine renal MT; and Class III: atypical, non-translationally synthesized metal thiolate polypeptides [23]). The family of metallothionein-like proteins with a carboxy-terminal (hereafter “C-terminal”) Gly was characterized for the first time in the yeast *Schizosaccharomyces pombe* exposed to cadmium [26]. Shortly thereafter, similar peptides were found in several plants exposed to various heavy metals. Phytochelatins have a structural relationship to glutathione. Homologues related to homoglutathione were called homophytochelatins and those related to hydroxymethylglutathione were designated as hydroxymethylphytochelatins. Those peptides having a C-terminal amino acid other then Gly are called isophytochelatins with the parenthetic addition of the C-terminal amino acid. The prefix iso- was chosen to signify the peptides based on the common structural element as γ-GluCys. Specific thiols are named according to their sequence of amino acids [27,28,29,30,31,32]. Plants carrying the MT gene also have been prepared as a way of increasing the ability of a plant to withstand metal ions [33,34,35,36,37,38,39,40].

The aim of this study was to investigate the effect of mold on low molecular mass thiols in grasses. For this purpose, the three grass species *Lolium perenne*, *Festulolium pabulare*, and *Festulolium braunii* were cultivated and sampled during four months, from June to September. High-performance liquid chromatography with electrochemical detection (HPLC-ED) was used for quantification of thiols—cysteine, reduced and oxidized glutathione, and phytochelatins (PC2, PC3, PC4 and PC5). The data were statistically processed and analyzed.

## 2. Experimental Section

### 2.1. Chemicals

Cysteine (CYS), reduced (GSH) and oxidized (GSSG) glutathione, and trifluoroacetic acid (TFA) were purchased from Sigma-Aldrich (St. Louis, MO, USA). Phytochelatins: (γ-Glu-Cys)2-Gly phytochelatin2 (PC2), (γ-Glu-Cys)3-Gly phytochelatin3 (PC3), (γ-Glu-Cys)4-Gly phytochelatin4 (PC4), and (γ-Glu-Cys)5-Gly phytochelatin5 (PC5) were synthesized at Clonestar Biotech (Brno, Czech Republic) with a purity above 90%. HPLC-grade methanol (>99.9%; *v*/*v*) came from Merck (Dortmund, Germany). Other chemicals were purchased from Sigma-Aldrich, unless noted otherwise. Stock standard solutions of the thiols (1 mg/mL) were prepared with ACS water (Sigma-Aldrich) and stored in darkness at −20 °C. Working standard solutions were prepared daily by dilution of the stock solutions. All solutions were filtered through 0.45 μm nylon filter discs (Millipore, Billerica, MA, USA) prior to analysis using HPLC-ED. The pH was measured using an inoLab WTW Level 3 instrument equipped with a Level 3 probe (inoLab, Weilheim, Germany).

### 2.2. Plant Material and Cultivation

#### 2.2.1. Description of the Site

A small-plot experiment was conducted at the Research Station of Fodder Crops in Vatin, Czech Republic (49° 31’ N, 15° 58’ E) at an altitude of 560 m a.s.l. The soil type used in the experiment was cambisol as sandy loam on a diluvium of biotic orthogneiss. The mean annual temperature in 2011 was 7.4 °C. The soil nutrient content was composed of 89.1 mg/kg P, 231.6 mg/kg K, and 855 mg/kg Ca, and the pH was 4.76. The plot experiment (field experiment) was carried out under natural conditions of the Czech–Moravian Highlands.

#### 2.2.2. Experimental Plots

*Lolium perenne*, *F. pabulare*, and *F. braunii* were used in the experiment. Grass species (S) was the first experimental factor. A split-plot design was used with plots of 1.5 × 10 m. The experiment was carried out in three independent replications. Evaluation was conducted at the stands in the fourth harvest year. The plots were fertilized with 50 kg/ha N in the spring, while during the experiment the plants were not fertilized. There were three harvest dates (second experimental factor: date of sample, D): 7 June 2011, 1 August 2011, and 23 September 2011. Treatment with the fungicide Amistar (active ingredient azoxystrobin, 250 g l^-1^) was carried out on 28 June and 13 July. The dose of Amistar was 3 l/ha. Application (Treatment, T) or non-application (without fungicide, control) of fungicide was the third experimental factor.

#### 2.2.3. Control Pot Experiment

The biomass of species evaluated from the field experiment was compared with that of these species grown under controlled conditions in a regime with conditions of 20 °C day (12 h), 10 °C night (12 h), and relative humidity 65%. The studied plant species (S) (*L. perenne, F. pabulare*, and *F. braunii*) were grown in vegetative pots of size 530 × 360 × 360 mm. Lawn soil (peat, compost, silica sand, mineral compound fertilizer N, P, K, trace elements, and finely ground dolomitic limestone applied at a rate equivalent to 40 kg/ha) was used as the soil substrate. Biomass sampling was performed 60 d after sowing.

#### 2.2.4. Description of the Species

*Lolium perenne* is a species susceptible to fungal diseases. This species is demanding of moisture and nutrients. Because of its vulnerability to freezing, stand perseverance in Central Europe is limited to a period of 4–6 years.

*Festulolium pabulare*, resulting from crossbreeding *Festuca arundinacea* and *Lolium multiflorum*, shows higher resistance to fungal diseases. This species has short rhizomes, is resistant to drought, and capable of growing under low temperatures. Leaves dry easily in autumn and are not so susceptible to putrefactive processes. It is a perennial species.

*Festulolium braunii* results from crossbreeding *Festuca pratensis* and *Lolium multiflorum*. This species produces excellent quality forage, but is similar to *L. perenne* in its susceptibility to fungal diseases, although resistance to *Fusarium* was increased by crossing with *Festuca pratensis*. Stand perseverance is limited to five years.

### 2.3. Description of Fungal Disease Identification and Assessment

Fungi species were identified based upon cultivation on specific media and specific metabolic markers. The infection was assessed on a scale from 1 (highest level of infection) to 9 (plots free of disease). Degree 1 represents very low resistance to fungal diseases (>90% diseased plant leaves per plot), degree 3 low resistance (65–90% diseased plant leaves per plot), degree 5 intermediate resistance (40–65% diseased plant leaves per plot), degree 7 high resistance (15–40% diseased plant leaves per plot), and degree 9 very high resistance (<15% diseased plant leaves per plot). Evaluated on two dates (31 May and 12 July), the levels of pathogen infection in experimental plots ranged on the 9-point scale from 7.3 to 8.0.

### 2.4. Sample Preparation for Fresh Weight Analysis

The plants were sampled at specific time intervals. Immediately after sampling, the plants were washed three times with distilled water, dried with filter paper, and frozen using liquid nitrogen prior to further experimental procedures. To determine fresh weight, the plants were weighed on Sartorius R160P balances (Sartorius GmbH, Goettingen, Germany) immediately after collection from the filter paper. 

### 2.5. Sample Preparation for Low Molecular Mass Determination

Weighed plant tissues (approximately 0.2 g fresh weight) were transferred to a test tube and prepared according to Supalkova *et al.* [41]. Liquid nitrogen was then added to the test tube, and the samples were frozen to disrupt the cells. Subsequently, 1 mL of 0.2 M phosphate buffer (pH 7.2) was added to the test tube. The mixture was prepared using an ULTRA-TURRAX T8 hand-operated homogenizer (IKA, Staufen, Germany) at 25,000 rpm for 3 min. Homogenate was then transferred to a new test tube, where it was shaken on a Vortex-2 Genie (Scientific Industries, New York, NY, USA) at 4 °C for 30 min. The homogenate was centrifuged (14,000 g) for 30 min at 4 °C using a Universal 32 R centrifuge (Hettich-Zentrifugen GmbH, Tuttlingen, Germany). The supernatant was filtered through a 0.45 μm nylon membrane filter disk (Millipore, Billerica, MA, USA) prior to analysis.

### 2.6. Determination of Low Molecular Mass Thiols

The HPLC-ED system consisted of two solvent delivery pumps (Model 582, ESA Inc., Chelmsford, MA, USA) operating in the range of 0.001–9.999 mL/min, a Zorbax eclipse AAA C_18_ (150 × 4.6; 3.5 μm particles, Agilent Technologies, Palo Alto, CA, USA) column and a CoulArray electrochemical detector (Model 5600A, ESA Inc.). The electrochemical detector included one flow cell (Model 6210, ESA Inc.). The cell consisted of four working porous carbon electrodes, each with auxiliary and dry Pd/H_2_ reference electrodes. Both the detector and the reaction coil/column were thermostated at 32 °C. The sample (20 μl) was injected using an autosampler (Model 542 HPLC, ESA Inc.). Samples were kept in the carousel at 8 °C during the analysis. The mobile phase consisted of 80 mM TFA (A) and methanol (B). The compounds of interest were separated by the following linear gradient: 0 → 1 min (3% B), 1 → 2 min (10% B), 2 → 5 min (30% B), and 5 → 6 min (98% B). The mobile phase flow rate was 1 ml/min, and the working electrode potential was 900 mV [42,43,44,45,46]. Time of analysis was 30 min.

### 2.7. Descriptive Statistics

Data were processed using Microsoft Excel^®^ (Microsoft Corporation, Redmond, WA, USA) and Statistica CZ Version 8.0 (Prague, Czech Republic). Results are expressed as mean ± standard error of the mean, unless noted otherwise (Excel^®^). Statistical significances were determined using Statistica CZ. Differences with *p* < 0.05 were considered significant and were determined using multifactorial ANOVA (in particular, Scheffé’s test), which was applied for comparing means.

## 3. Results and Discussion

### 3.1. Mold Influence on Plants

**Figure 1:**
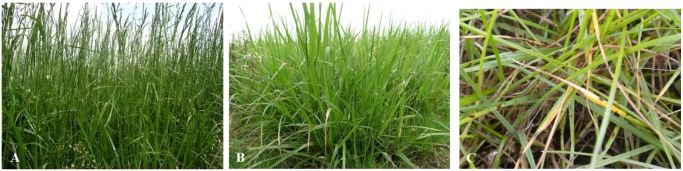
(**A**) *Festulolium pabulare* vegetation before the first cutting. (**B**) Leaf shoots of *Festulolium pabulare* before the second cutting in untreated plants. (**C**) *Puccinia* sp. on leaves of *Lolium perenne*.

In this study, three grass species—*F. pabulare *(Figure 1A and B)*, L. perenne* (Figure 1C) and *F. braunii*—were studied to determine whether low molecular mass thiols are involved in protective mechanisms against molds in grasses. Detecting the presence of molds on the species during the cultivation period was of primary interest. It is common knowledge that grasses can be attacked by many species of fungi. 

In the spring of 2011, there was widespread infection of grasses by *Fusarium nivale*. Additionally, many plants experienced multiple infections by *Colletotrichum graminicola*, *F. pabulare* and *Ascochyta*. In late summer and autumn, *Puccinia graminis* and *Puccinia coronata* occurred on all experimental species (Figure 1C). To a lesser extent, *Blumeria graminis* was also detected. Such other fungi as *Alternaria* spp., *Cochliobolus* spp., *Aspergillus niger*, and *Ramularia* spp. also were observed, but their occurrence was negligible as compared to the aforementioned species. Fungal diseases altered forage production (volume) and quality due to the fact that grasses actively defend themselves against the presence of a pathogen. There are two main types of defense pathways: (i) mechanical, and (ii) biochemical. Mechanical defense is based on an increase in the content of solid fibrous elements, which subsequently reduces the digestibility of organic matter and leads to a decline in forage quality [47]. The stress-induced biochemical pathways involve the production of several types of compounds which are able to detoxify the xenobiotics produced by fungi. Such low molecular mass thiols as cysteine, reduced glutathione, and phytochelatins are among these compounds. Thiols have been shown able to detoxify both inorganic [48,49,50,51,52] and organic [53,54,55,56,57] xenobiotics. One may also consider the fact that the occurrence of fungi may be associated with the risk of mycotoxins which reduce the health safety of forage [2]. Therefore, we were interested in evaluating the behavior of grasses attacked by a pathogen and the influence of treatment and mold presence on the content of low molecular mass thiols, because treatment itself by this fungicide, and other strobilurins, increases yield and delays plant senescence even in the absence of disease [58,59,60]. Respiration and the plant redox status are critical to the production of the thiols [61], and thus the potential for the fungicide treatment to alter this redox status is of great significance [62,63]. The fungicidal activity of strobilurins relies on their ability to inhibit mitochondrial respiration by binding at the so-called Qo site (the outer quinol-oxidation site) of the cytochrome *bc1* enzyme complex (complex III). This inhibition blocks electron transfer between cytochrome *b* and cytochrome *c1*, which, in turn, leads to an energy deficiency in fungal cells by halting the production of ATP [64]. Mitochondrial glutathione, as a key survival antioxidant, could serve in the forming of some resistance of fungi against these fungicides [65].

### 3.2. Plant Development

**Figure 2:**
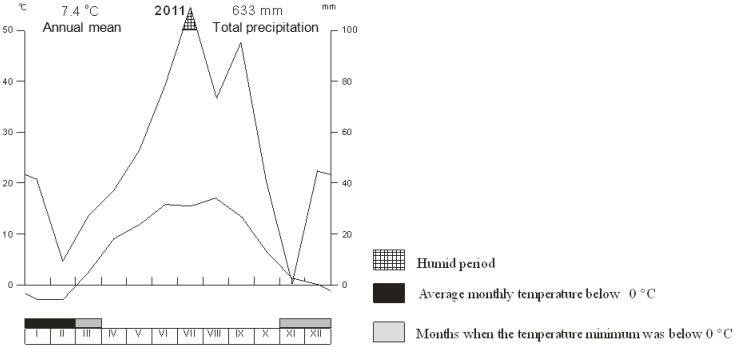
Temperature and precipitation at the Vatin research station in 2011.

Attention was primarily focused on plant development. Growth of the grasses was accompanied by a lengthening stalk formation and generative organs in the first crop. At the time of harvest, grasses were at earring stage. After the first cutting, a period of intense tillering followed. As shown in Figure 2, temperature and precipitation at the research station in Vatin were sufficient for growth within the period from June to July. An absence of vernalization was reflected in the formation of leafy shoots and absence of fertile stalks. Similar conclusions apply also for grass growth after the second cutting. No visual difference (growth, color) was present in the growing of plants treated with fungicide as compared with those not treated. Assessment of disease infection level of the studied species prior to and after the first fungicide application is shown in Table 1. While during the summer months the species showed comparable levels of disease infection, that situation changed somewhat when stronger pathogen pressure was apparent in late September. No visible differences in infection level were found between grasses treated with fungicides and those untreated. A tendency toward greater pathogen pressure was evident in *L. perenne*, but the differences shown in Table 1 were not statistically significant, even though *L. perenne* is a species more susceptible to pathogens as compared to the more resistant *Festuca arundinacea *and its hybrids. [66,67]. Plants in the experiment were not artificially infected, and the pathogens did not occur. The level of infection can be influenced by many factors. Comparable and low levels of infection in the evaluated species could have been affected by weather conditions adverse for the growth of mold. Had higher pressure from pathogens been evident, that might have substantiated a benefit from use of fungicides and showed the a priori anticipated differences between treated and untreated variants. The plants sown under controlled conditions served as a control group and showed no occurrence of fungal diseases. Under the controlled conditions, grass sprouted between 4 d and 10 d. Due to the fact that these are winter species, they formed leafy shoots, with no tendency to produce fertile stalks. Biomass sampling was performed 60 d after sowing in the tillering stage.

**Table 1:** Level of infection in *Lolium perenne*, *Festulolium pabulare*, and *Festulolium braunii* before and after first fungicide application (on scale of 1 (highest infection) to 9 (lowest infection)).

Date evaluated	*Lolium perenne*		*Festulolium pabulare*		*Festulolium braunii*	
	No fungicide	Fungicide ^1^	No fungicide	Fungicide ^1^	No fungicide	Fungicide ^1^
31.5.2011	7.3	7.3	7.7	7.7	8.0	7.3
12.7.2011	7.3	7.7	8.0	8.0	8.0	8.0
26.9.2011	3.0	3.0	4.0	4.0	3.0	3.0

^1 ^Fungicide was applied on 28 June 2011.

### 3.3. Low Molecular Mass Thiols

Next, low molecular mass thiols were determined. Determination was carried out as in our previously published work [42,43,44,45,46], in which our group had demonstrated detection limits for these thiols measurable down to nanograms per gram of fresh material (ng/g), with a linear detection range down to four orders of magnitude in concentration and recovery of the thiols exceeding 80%. This method was used to analyze the experimental plants. GSH and GSSG are well-known markers of oxidative stress and therefore can be used as good markers of the negative effect of mold on grasses. In relation to phytochelatins, they are mainly considered as metal pollution markers, but their richness in thiol moieties could give them the ability to scavenge reactive oxygen species [68,69]. *Festulolium pabulare* had higher levels of GSH and PC3 in the pot experiment (Table 2). Moreover, lower GSSG content resulted in a high GSH/GSSG ratio.

**Table 2:** Effect of Species (S) on contents of CYS, GSH, GSSG, PC2, PC3, PC4, PC5, and GSH/GSSG ratio in pots experiment. Content of CYS, GSH and GSSG is expressed as µg/g of fresh weight. PC2, PC3, PC4, and PC5 are expressed as ng/g of fresh weight.

	Factor							
Species (S)	CYS	GSH	GSSG	GSH/GSSG	PC2	PC3	PC4	PC5
*Lolium perenne*	0.65	2.16	2.16	1.00	0.239	1.42	0.74	1.02
*Festulolium pabulare*	1.43	3.23	0.43	7.56	0.28	3.82	0.44	0.05
*Festulolium braunii*	6.27	1.71	1.78	0.96	0.47	0.64	0.30	0.03

In the field experiment (Table 3 and Table 4), PC2, PC4, and PC5 content was higher in *F. pabulare*. Conversely, *F. braunii* showed higher content of CYS. The species differed significantly (*p* < 0.05), however, in PC4 and PC5 content. Lower contents of GSH and GSSH and a lower GSH/GSSG ratio were recorded in September. The differences between months were not statistically confirmed. An exception was the low content of GSSG (*p* < 0.05) in September, taking into account the treatment factor (Table 4). Fungicide application did not result in demonstrable differences in the average content of low molecular mass thiols (Table 4).

**Table 3:** Effect of Species (S) and Date of sample (D) on contents of CYS, GSH, GSSG, PC2, PC3, PC4, PC5 and GSH/GSSG ratio in field experiment. Content of CYS, GSH and GSSG is expressed as µg/g of fresh weight. PC2, PC3, PC4 and PC5 are expressed as ng/g of fresh weight.

	Factor							
	CYS	GSH	GSSG	GSH/GSSG	PC2	PC3	PC4	PC5
**Species (S)**								
*Lolium perenne*	2.9 ± 1.6	8.3 ± 4.5	1.5 ± 0.9	5.5 ± 2.5	0.5 ± 0.3	0.4 ± 0.1	0.6 ± 0.3^a^	0.8 ^a ^± 0.4
*Festulolium pabulare*	6.2 ± 1.7	9.0 ± 5.0	4.7 ± 1.8	1.7 ± 0.4	0.5 ± 0.1	0.5 ± 0.2	2.7 ± 0.3^b^	2.8 ^a ^± 1.1
*Festulolium braunii*	8.2 ± 3.9	7.4 ± 2.3	2.9 ± 1.1	3.3 ± 1.5	0.5 ± 0.3	0.5 ± 0.3	1.4 ± 0.2^a^	0.3 ^a ^± 0.2
**Date of sample (D)**								
June	7.1 ± 0.8	11.3 ± 3.8	3.4 ± 2.4	6.2 ± 2.2	0.3 ± 0.1	0.2 ± 0.1	1.6 ± 1.0	0.6 ± 0.2
August	2.5 ± 0.3	11.0 ± 3.2	4.1 ± 0.5	2.9 ± 1.1	0.7 ± 0.3	0.5 ± 0.2	1.3 ± 0.5	2.0 ± 1.0
September	7.6 ± 4.4	2.3 ± 0.8	1.7 ± 0.6	1.4 ± 0.1	0.5 ± 0.3	0.6 ± 0.3	1.9 ± 0.4	1.3 ± 1.3

Average values with superscripts (^a,b^) are significant at *p* < 0.05 and *p* < 0.01, respectively*.*

**Table 4:** Effect of Species (S), Date of sample (D), and Treatment (T) on contents of CYS, GSH, GSSG, PC2, PC3, PC4, PC5 and GSH/GSSG ratio in field experiment. Content of CYS, GSH and GSSG is expressed as µg/g of fresh weight. PC2, PC3, PC4 and PC5 are expressed as ng/g of fresh weight.

	Factor							
	CYS	GSH	GSSG	GSH/GSSG	PC2	PC3	PC4	PC5
**Species (S)**								
*Lolium perenne*	1.8 ± 0.6	7.0 ± 3.5	2.3 ± 0.6	2.6 ± 0.9	0.7 ± 0.2	0.5 ± 0.1	1.8 ± 0.7	0.7 ± 0.3 ^a^
*Festulolium pabulare*	4.8 ± 1.1	4.2 ± 0.6	2.8 ± 0.7	2.0 ± 0.8	1.2 ± 0.5	0.5 ± 0.1	3.7 ± 1.4	2.6 ± 0.9 ^b^
*Festulolium braunii*	9.7 ± 4.9	9.2 ± 3.7	3.1 ± 0.7	2.8 ± 1.0	0.7 ± 0.2	0.8 ± 0.2	1.2 ± 0.3	0.3 ± 0.2 ^a^
**Date of sample (D)**								
August	6.2 ± 2.9	9.8 ± 2.7	3.6 ± 0.4^a^	2.8 ± 0.8	0.8 ± 0.2	0.6 ± 0.1	2.7 ± 1.1	1.2 ± 0.6
September	4.6 ± 2.4	3.8 ± 1.0	1.9 ± 0.4^b^	2.1 ± 0.5	1.0 ± 0.3	0.6 ± 0.1	1.8 ± 0.3	1.2 ± 0.7
**Treatment (T)**								
No fungicide	5.0 ± 2.3	6.6 ± 2.4	2.9 ± 0.6	2.1 ± 0.6	0.6 ± 0.2	0.6 ± 0.1	1.6 ± 0.3	1.7 ± 0.7
Fungicide	5.8 ± 3.0	7.0 ± 2.5	2.6 ± 0.5	2.8 ± 0.8	1.2 ± 0.3	0.6 ± 0.1	2.9 ± 1.1	0.7 ± 0.4

Average values with superscripts (^a,b^) are significant at p < 0.05 and p < 0.01, respectively.

A few papers reporting on the evaluation of thiol content in the biomass of grasses have been published. For example, Raab *et al.* detected PC2 and PC3 in the biomass of *Holcus lanatus* [70]. This species is resistant to heavy metals and its arsenic contamination showed a higher content of PC3 than did *Pteris cretica*, which is vulnerable to heavy metals. In our pots experiment (Table 2), disease-resistant *F. pabulare* showed higher PC3 levels than did the more pathogen-susceptible *L. perenne* and *F. braunii*. In our field experiment, the content of PC4 and PC5 was significantly higher also in *F. pabulare*. Hunaiti *et al.* evaluated the influence of heavy metals and sulfate content on thiols in *Cynodon dactylon*, *Amaranthus gracilis*, *Chenopodium murale*, and *Typha domingensis* [71]. The increase of metal and sulfate levels in the soil at their contaminated site was correlated with a rise of total glutathione and cysteine in plants. However, the individual effects of metals and sulfate on glutathione, short-chain phytochelatin, and long-chain phytochelatin levels were dissimilar. According to Jia *et al.*, this fact depends on plant species [72].

### 3.4. Interactions

**Figure 3:**
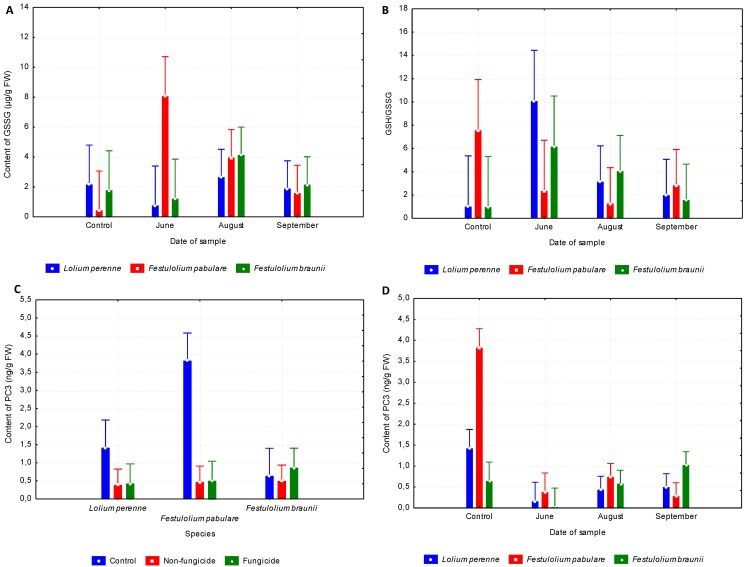
(**A**) GSSG content in relation to Date of sample and Species. (**B**) GSH/GSSG ratio in relation to Date of sample and Species. (**C**) PC3 content in relation to Species and Treatment. (**D**) PC3 content in relation to Date of sample and Species.

The content of GSSG found in the pots experiment was comparable to the values determined under field conditions in *L. perenne* and *F. braunii* during the growing season. That assessment does not apply to *F. pabulare*, where an obvious decrease of GSSG from June to September and low GSSG content in plants grown under control conditions was observed (Figure 3A). The relationship between Date and Species was significant (*p* < 0.05) in the case of GSSG (Table 5). Comparable small changes in GSSG content during the growing season are reported in those species susceptible to fungal diseases (*i.e.*, *L. perenne* and *F. braunii*). In contrast, *F. pabulare*, which can be considered resistant to infection, clearly showed a high GSSG content in June. Moreover, the content of GSSG in *F. pabulare* was low under control conditions.

**Table 5:** Relationship between Date of sample (D) and Species (S) and between Species (S) and Treatment (T).

Factor	CYS	GSH	GSSG	GSH/GSSG	PC2	PC3	PC4	PC5
D × S	0.9915	0.4084	0.0393	0.0750	0.9928	0.0005	0.8305	0.5962
S × T	0.9835	0.9008	0.5880	0.2044	0.5229	0.0017	0.4169	0.5111

Similarly, differences between species susceptible to fungal diseases (*L. perenne* and *F. braunii*) and resistant species (*F. pabulare*) in the assessment of GSH/GSSG are shown in Figure 3B. While the more susceptible species show a higher ratio of GSH/GSSG in June, decreasing thereafter in the continuing growing season, *F. pabulare* shows comparable values from June to September. A higher ratio of GSH/GSSG was found, however, in plants of *F. pabulare* grown under control conditions. Biomass obtained from plants grown under controlled conditions had a higher content of PC3. This fact, however, applied only to *L. perenne* and (especially so) to *F. pabulare *(Figure 3C). The relationship between Species and Treatment was significant (*p* < 0.05, Table 5). Even more substantial are the differences between PC3 values. *Lolium perenne* and *F. braunii* show comparable PC3 content from June to September. *Lolium perenne* grown under control conditions exhibited a higher PC3 content, but this discrepancy was not statistically significant. Conversely, *F. pabulare* grown in control conditions had high (*p* < 0.05) PC3 content (Figure 3D). Differences in PC3 content between plants grown under control conditions and in field experiments are very obvious. Biomass obtained from plants grown under controlled conditions had a higher PC3 content. This fact, however, applies only to L. *perenne* and (especially so) to *F. pabulare*. The relationship between Species and Treatment was significant (*p* < 0.05, Table 5).

## 4. Conclusions

Low molecular mass thiols represented by glutathione and phytochelatins are well-known markers of metal intoxication in plants, as their synthesis is catalyzed by the presence of metal ions in the intracellular environment [73]. Based on recently published studies, associations with some other types of stress, including from organic pollutants, have been indicated [55,57]. This study showed that the association of thiol content and the presence of pathogens should be taken into account. The enhancement of PC3 is interesting, and one may speculate that this thiol might be somehow involved mainly in detoxification of pathogen toxins through certain reactive oxygen scavenging pathways. This must be confirmed and clarified, however, by follow-up greenhouse and field experiments.
